# Contribution of the yeast bi-chaperone system in the restoration of the RNA helicase Ded1 and translational activity under severe ethanol stress

**DOI:** 10.1016/j.jbc.2023.105472

**Published:** 2023-11-17

**Authors:** Ryoko Ando, Yu Ishikawa, Yoshiaki Kamada, Shingo Izawa

**Affiliations:** 1 Graduate School of Science and Technology, Kyoto Institute of Technology, Sakyo-ku, Kyoto, Japan; 2 National Institute for Basic Biology, Okazaki, Aichi, Japan

**Keywords:** yeast, stress granule, protein denaturation, translation control, translation initiation factor, severe ethanol stress, acquired resistance, Ded1, bi-chaperone system

## Abstract

Preexposure to mild stress often improves cellular tolerance to subsequent severe stress. Severe ethanol stress (10% v/v) causes persistent and pronounced translation repression in *Saccharomyces cerevisiae*. However, it remains unclear whether preexposure to mild stress can mitigate translation repression in yeast cells under severe ethanol stress. We found that the translational activity of yeast cells pretreated with 6% (v/v) ethanol was initially significantly repressed under subsequent 10% ethanol but was then gradually restored even under severe ethanol stress. We also found that 10% ethanol caused the aggregation of Ded1, which plays a key role in translation initiation as a DEAD-box RNA helicase. Pretreatment with 6% ethanol led to the gradual disaggregation of Ded1 under subsequent 10% ethanol treatment in wild-type cells but not in *fes1*Δ*hsp104*Δ cells, which are deficient in Hsp104 with significantly reduced capacity for Hsp70. Hsp104 and Hsp70 are key components of the bi-chaperone system that play a role in yeast protein quality control. *fes1*Δ*hsp104*Δ cells did not restore translational activity under 10% ethanol, even after pretreatment with 6% ethanol. These results indicate that the regeneration of Ded1 through the bi-chaperone system leads to the gradual restoration of translational activity under continuous severe stress. This study provides new insights into the acquired tolerance of yeast cells to severe ethanol stress and the resilience of their translational activity.

The budding yeast *Saccharomyces cerevisiae* possesses a higher ethanol tolerance than other organisms and efficiently produces ethanol through alcoholic fermentation. However, high concentrations of ethanol are harmful to yeast cells due to diverse adverse effects, including depolarization of the actin cytoskeleton (1), perturbation of membrane fluidity and transport systems (2, 3, 4), and prevention of the nuclear export of bulk poly(A)^+^ mRNA (5, 6). Moreover, ethanol at high concentrations reduces the growth rate, viability, and fermentation ability of yeast cells (7, 8). Severe ethanol stress also causes protein denaturation and the formation of denatured protein deposits in yeast cells (9, 10, 11).

Translational activity is often repressed under various severe stress conditions (12, 13, 14, 15, 16, 17, 18). Severe acute ethanol stress (10% v/v) also causes persistent and pronounced translation repression in yeast cells (19, 20). Pronounced translation repression is often accompanied by the formation of processing bodies (PBs) and stress granules (SGs), which are cytoplasmic messenger ribonucleoprotein granules consisting of non-translating mRNAs and various proteins (21, 22, 23, 24, 25, 26, 27). Indeed, severe ethanol stress and glucose depletion cause translation repression with the formation of PBs and SGs in yeast cells (22, 28, 29, 30). PBs and SGs play important roles in regulating the cytosolic mRNA flux under stress conditions (27, 31, 32, 33, 34).

As one of the well-analyzed mechanisms for translation repression, phosphorylation of eIF2α by the protein kinase Gcn2 is known to prevent the regeneration of eIF2-GDP to eIF2-GTP by eIF2B, resulting in decreased levels of the ternary complex (eIF2-GTP Met-${\text{tRNA}}_{i}^{\text{Met}}$) (35, 36, 37, 38). As another mechanism of translation repression, the loss of RNA helicase activity has been reported to inhibit translation initiation. eIF4A (Tif1 and Tif2) and Ded1 are yeast RNA helicases that unwind the secondary structure at the 5′ end of mRNA for the assembly of the 43S pre-initiation complex (PIC) (16, 39). Hence, stress-induced inactivation of eIF4A and Ded1 prevents PIC binding to mRNA and stalls the start codon scanning process (40, 41, 42, 43). Moreover, eIF4A, Ded1, and eIF4B (Tif3) dissociate from mRNA during heat shock and glucose depletion (41). In particular, Ded1 has been reported to aggregate as a component of SGs under heat shock and glucose depletion (42, 44). So far, however, there has been no report on whether phosphorylation of eIF2α and/or inactivation of RNA helicases are involved in ethanol-induced translation repression in yeast cells.

Yeast cells acquire resistance to the various adverse effects of severe ethanol stress through pre-exposure to mild ethanol stress. Pre-exposure to mild ethanol stress improves yeast growth under subsequent exposure to high concentrations of ethanol (45, 46). Moreover, pretreatment with 6% (v/v) ethanol confers resistance to the protein denaturation caused by 10% (v/v) ethanol (10). The induced expression of stress-related proteins, such as heat shock proteins (HSPs), and changes in the lipid composition of the plasma membrane during preexposure to mild ethanol stress contribute to improved resistance to high concentrations of ethanol (10, 47, 48). However, it is currently unknown whether preexposure to mild ethanol stress mitigates translation repression under severe ethanol stress.

Therefore, in the present study, we examined whether preexposure to mild ethanol stress induced acquired resistance to severe ethanol stress-induced translation repression. We found that pretreatment with 6% ethanol could restore the translational activity of yeast cells after transient translation repression under subsequent 10% ethanol stress. We also confirmed that 10% ethanol induced the formation of Ded1 aggregates but not eIF2α phosphorylation. Moreover, pretreatment with 6% ethanol led to gradual disaggregation of Ded1-aggregates under severe ethanol stress. However, ethanol pretreatment did not sufficiently restore translational activity and induce disaggregation of Ded1 in *fes1Δhsp104*Δ cells, which are deficient in Hsp104 with a significantly reduced capacity of Hsp70. These results suggest that the regeneration of Ded1-aggregates through the bi-chaperone system plays an important role in the restoration of translational activity under severe ethanol stress. This study provides novel insights into the resilience of translational activity in yeast cells under ethanol stress.

## Results

### Pre-exposure to mild ethanol stress mitigates translation repression under severe ethanol stress

**Figure 1:**
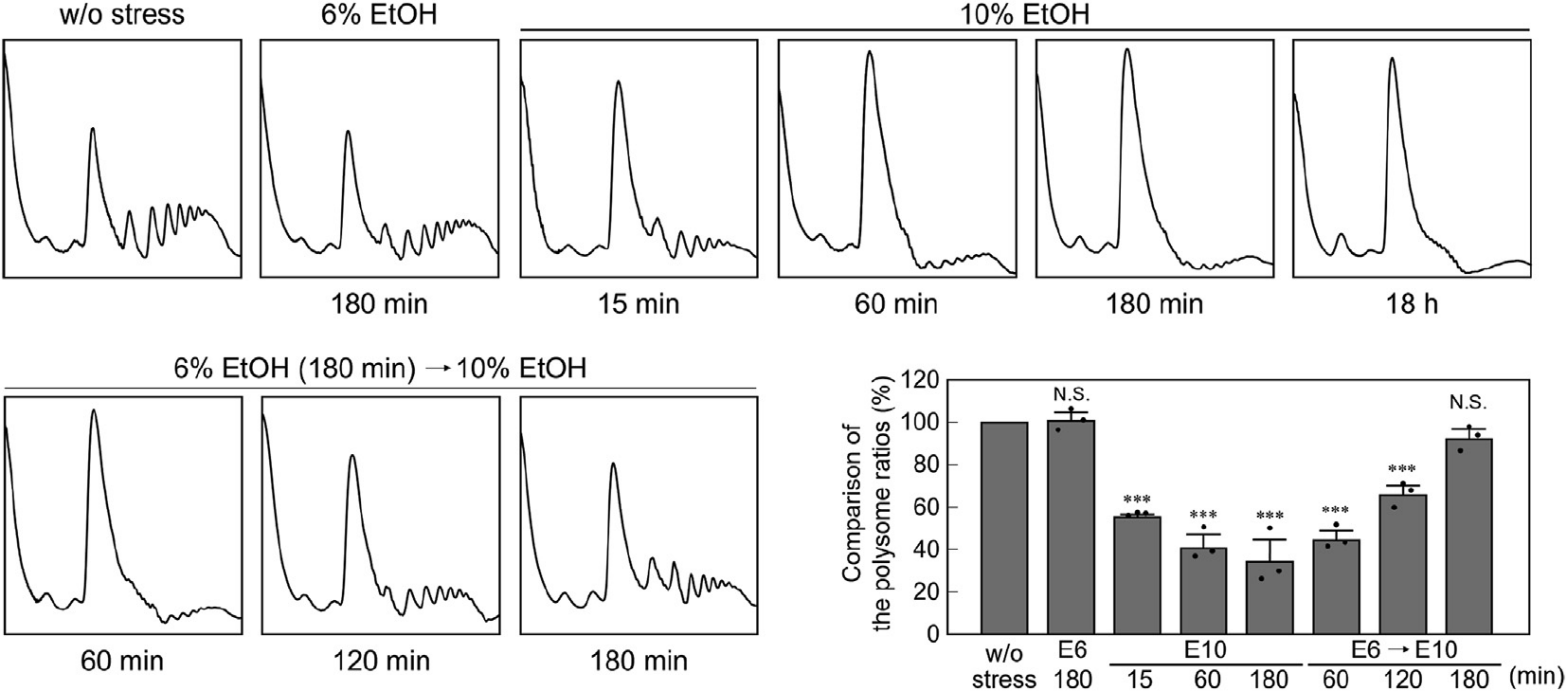
**Restoration of translational activity under severe ethanol stress in cells pre-exposed to mild ethanol stress.** The polysome profiles of the yeast cells under ethanol stress were determined. Cells were pre-exposed or not pre-exposed to mild ethanol stress (6% v/v, E6) for 180 min and then exposed to severe ethanol stress (10% v/v, E10). The polysome ratios (the area under polysomal ribosome peaks/the area under total ribosome peaks) under each condition were compared and expressed as a percentage of that of the non-stressed cells (mean ± S.D., *n* = 3). Significant differences were evaluated using Dunnett’s post-hoc test. ∗∗∗*p <* 0.001, N.S., statistically not significant.

In this study, as an indicator of translational activity, the polysome ratio (area under the polysomal ribosome peaks/area under the total ribosome peaks) was measured by polysome analysis. As previously reported (19), direct exposure of yeast cells to severe ethanol stress (10% v/v) quickly reduced the polysome ratios (*i.e.*, polysome peaks were quickly and significantly reduced and 80S monosome peaks were increased), suggesting that pronounced translation repression was induced (Fig. 1). The reduced polysome ratio persisted for at least 18 h under 10% ethanol stress. In contrast, exposure to mild ethanol stress (6% v/v) caused little reduction in the polysome peaks.

Next, we examined whether pretreatment with 6% ethanol affected translational activity under subsequent severe ethanol stress. When yeast cells were pretreated with 6% ethanol for 180 min and then challenged with 10% ethanol, pronounced translation repression was initially induced, but a gradual recovery of the polysome ratios was then observed (Fig. 1). The pretreated cells recovered approximately 90% of their original polysome ratio after 180 min, even in the presence of 10% ethanol. These results indicated that yeast cells acquired the ability to restore the polysome ratio under severe ethanol stress following preexposure to mild ethanol stress.

**Figure 2:**
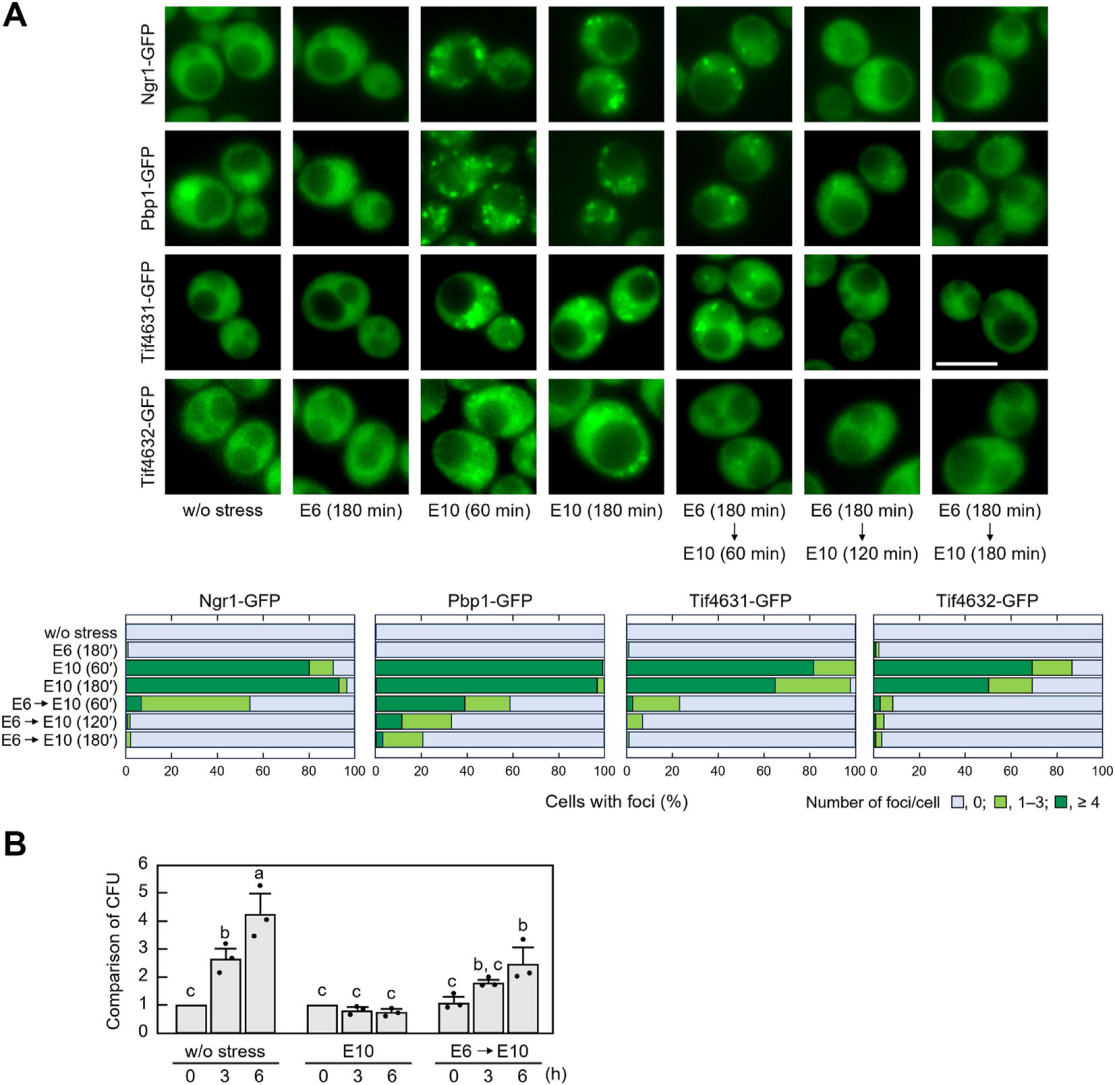
**Disassembly of SGs and recovery of proliferation under severe ethanol stress in wild-type cells pre-exposed to mild ethanol stress**. *A*, SG formation was examined using GFP-tagged Ngr1, Pbp1, Tif4631, and Tif4632 under the stress conditions indicated. Representative images are shown in the *upper panels*, and quantified data are shown in the *lower panels*. Experiments were independently repeated three times and more than 300 cells in total were examined. Scale bar, 5 μm. *B*, cell proliferation was evaluated by the colony-forming unit (CFU) after the stress treatments. Yeast cells were pre-exposed or not pre-exposed to mild ethanol stress (6% v/v, E6) for 180 min and then exposed to severe ethanol stress (10% v/v, E10) for 0, 3, or 6 h. The CFU of cells w/o stress treatment at 0 h was set to a relative value of 1 (mean ± S.D., *n* = 3). Different letters indicate statistically significant differences (*p* < 0.05, ANOVA with post-hoc Tukey’s test).

We further examined whether the recovery of the polysome ratio reflected the recovery of translational activity. As pronounced translation repression is often accompanied by the formation of SGs, consisting of non-translating mRNAs and a variety of translation-related factors and ribosomal components (22, 23, 30, 49), we examined SG formation in the pretreated cells. GFP-tagged Ngr1, Pbp1, and eIF4G (Tif4631 and Tif4632) were used as SG markers in the analysis (22, 49). As reported previously (29), SG formation was clearly induced by direct exposure of yeast cells to 10% ethanol, whereas pretreatment with 6% ethanol resulted in alleviation of SG formation after 180 min of treatment with 10% ethanol (Fig. 2*A*). Additionally, the recovery of cell proliferation under 10% ethanol stress was also observed in the pretreated cells, but not in cells directly exposed to 10% ethanol (Fig. 2*B*). As cell proliferation requires new protein synthesis, these results suggest that cells pretreated with 6% ethanol recovered their translational activity under severe ethanol stress. Therefore, we interpreted the recovery of the polysome ratio in the pretreated cells as a reflection of the recovery of translational activity under severe ethanol stress.

No significant reduction in ethanol concentrations was observed in the cultures after treatment with 6% and 10% ethanol for 3 h (Fig. S1), confirming that the ethanol stress was not reduced *via* yeast metabolism.

### Severe ethanol stress induces changes in the subcellular localization of the scanning-related factors, Ded1 and eIF4B

**Figure 3:**
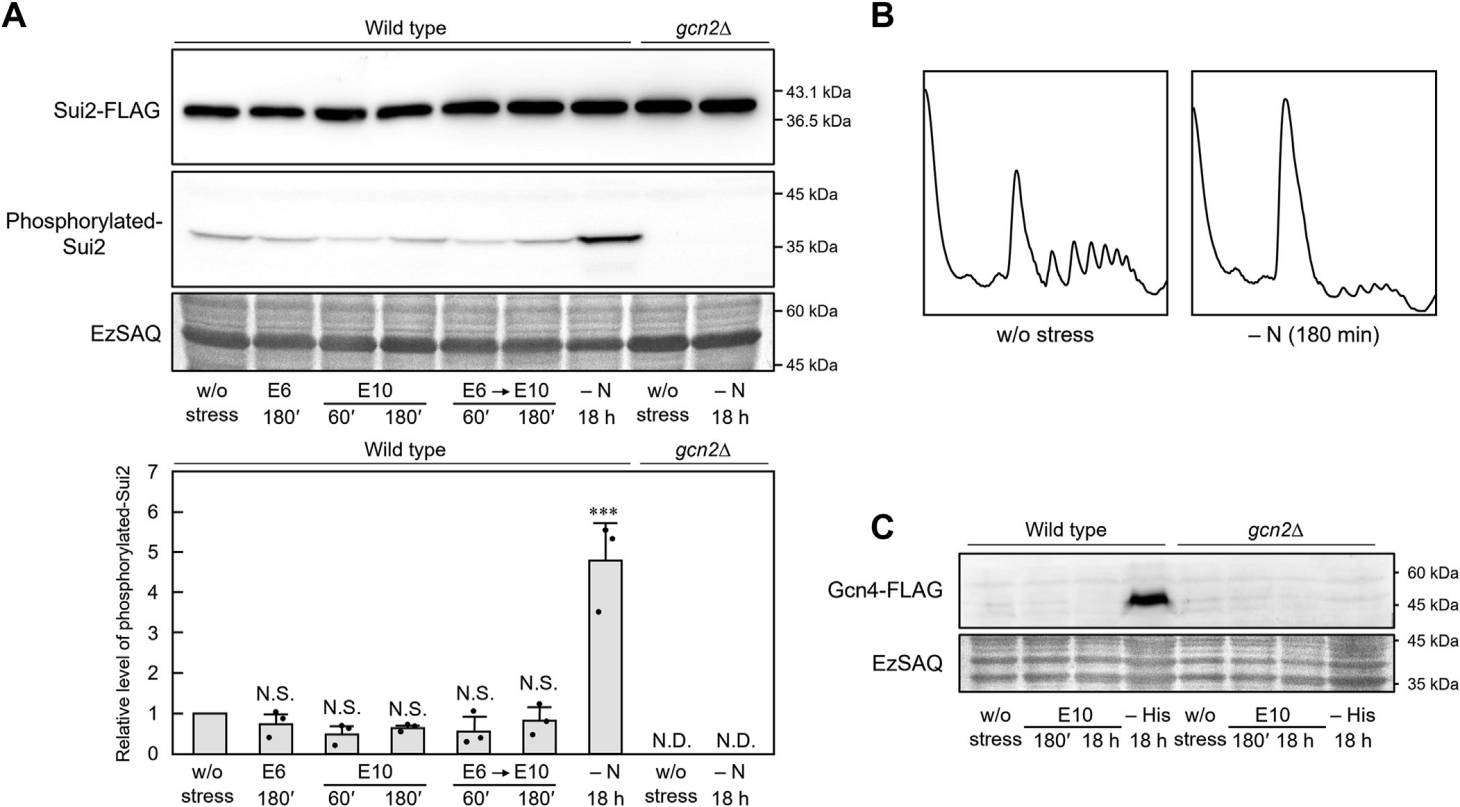
**Phosphorylation of eIF2α (Sui2) is not induced by severe ethanol stress**. *A*, yeast cells expressing Sui2-FLAG were pre-exposed or not pre-exposed to mild ethanol stress (6% v/v, E6) for 180 min and then exposed to severe ethanol stress (10% v/v, E10). Cells were also subjected to nitrogen starvation stress (– N) for 18 h. The levels of FLAG-tagged Sui2 and phosphorylated Sui2 were assayed *via* western blotting. The phosphorylated Sui2 levels were quantified using the ImageJ software. The phosphorylated Sui2 level of cells w/o stress treatment was set to a relative value of 1 (mean ± S.D., *n* = 3). Statistical significance was evaluated using Dunnett’s post-hoc test. ∗∗∗*p <* 0.001, N.S., statistically not significant. N.D., not detected. *B*, polysome analysis data of wild-type cells exposed to nitrogen starvation stress for 180 min. *C*, cells carrying *GCN4*-*FLAG* were exposed to severe ethanol stress (10% v/v, E10) for 180 min and 18 h. As a positive control, cells were also subjected to histidine starvation stress (–His) for 18 h. Gcn4-FLAG was not detected except in wild-type cells subjected to (–His). EzSAQ staining was performed to confirm equal loading and the transfer of all proteins.

To understand the mechanism underlying the recovery of translational activity in the pretreated cells subjected to 10% ethanol, we focused on the translation initiation stage. Phosphorylation of eIF2α is considered to be a major cause of translation repression in eukaryotic cells (50), and Gcn2 is the sole protein kinase responsible for the phosphorylation of eIF2α in *S. cerevisiae* (51). We first examined the effects of ethanol on eIF2α (Sui2) phosphorylation. As reported previously (52), we confirmed that eIF2α was phosphorylated in a protein kinase Gcn2-dependent manner under nitrogen starvation, which caused pronounced translation repression (Fig. 3, *A* and *B*). However, no change in the phosphorylation state of eIF2α due to ethanol stress was observed significantly even under treatment with 10% ethanol for 18 h (Figs. 3*A* and S2). These results suggest that phosphorylation/dephosphorylation of eIF2α is not closely involved in the suppression and recovery of translational activity under severe ethanol stress.

Synthesis of Gcn4, a transcriptional activator of amino acid biosynthetic genes, is selectively induced after eIF2α phosphorylation due to nitrogen source depletion (53). In contrast, fusel alcohols including butanol have been reported to cause *GCN4* translation by directly targeting eIF2B without mediating eIF2α phosphorylation (13). We examined whether ethanol had the same effect as fusel alcohols and found that ethanol treatment did not induce Gcn4 expression (Fig. 3*C*). Ethanol and fusel alcohols appeared to have different effects on eIF2B.

**Figure 4:**
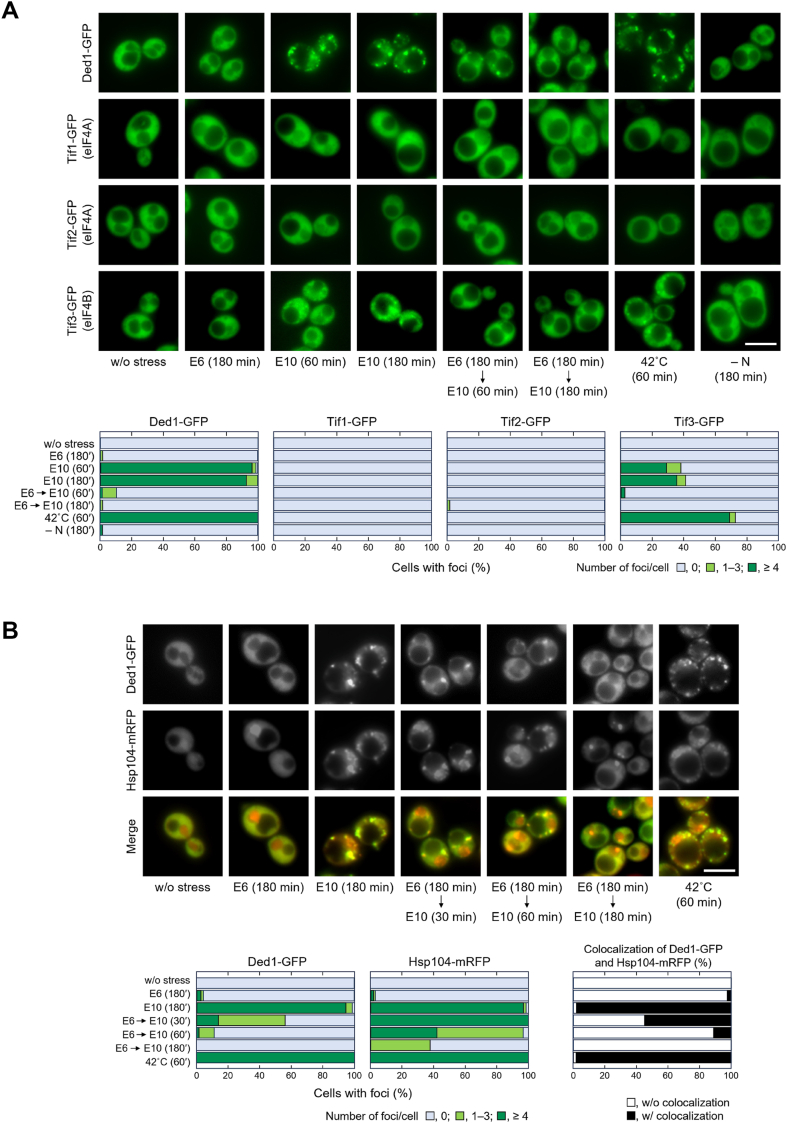
**Severe ethanol stress, as well as heat shock, causes the formation of Ded1 aggregates**. *A*, intracellular localization of the scanning-related factors was examined using yeast cells expressing GFP-tagged Ded1, eIF4A (Tif1 and Tif2), or eIF4B (Tif3) under the stress conditions indicated. *B*, colocalization of Ded1 and Hsp104 was monitored using wild-type cells expressing Ded1-GFP and Hsp104-mRFP. E6, E10, and – N mean 6% ethanol, 10% ethanol, and nitrogen starvation stress, respectively. Representative images are shown in the *upper panels*, and quantified data are shown in the *lower panels*. Experiments were repeated three times and more than 300 cells in total were examined. Scale bar, 5 μm.

Since eIF2α phosphorylation is not always required for repressing global translation under stress conditions (54), it was speculated that other causes of translation repression exist. Next, we examined the ethanol stress response of Ded1, one of the scanning-related factors. Ribosomal scanning is a key process in translation initiation, and it has recently been reported that Ded1 plays a role in translation regulation through dynamic changes in the stress response, that is, Ded1 dissociates from mRNA and aggregates in the cytoplasm following heat shock and glucose depletion, thereby inhibiting translation initiation (41, 42, 43). We examined the subcellular localization of Ded1-GFP and found that it aggregated and formed cytoplasmic granules under 10% ethanol, as well as under heat shock at 42 °C (42) (Fig. 4*A*). In contrast, 6% ethanol and nitrogen starvation did not cause the formation of Ded1 aggregates. Moreover, in cells pretreated with 6% ethanol, the formation of Ded1 granules was observed just after subjecting the cells to 10% ethanol stress, but these granules gradually disappeared with time, suggesting that the pretreated cells acquired the ability to restore Ded1 under severe ethanol stress (Fig. 4*A*).

We also found that 10% ethanol stress caused the aggregation of eIF4B (Tif3) (Fig. 4*A*), which is known to dissociate from mRNA during heat shock and glucose depletion (41). These results indicated that Ded1 and eIF4B dissociate from mRNA and condense under severe ethanol stress, similar to findings reported in relation to heat shock and glucose depletion (41, 42). In contrast, similar to glucose depletion and amino acid depletion (55), 10% ethanol and heat shock at 42 °C did not cause the aggregation of eIF4A (Tif1 and Tif2) (Fig. 4*A*).

Ded1 has also been reported to localize in SGs upon heat shock and glucose depletion (42, 44). We examined whether Ded1 colocalizes with Ngr1, a component of SGs (29). Because Ded1 granules colocalized with Ngr1 granules under severe ethanol stress as well as thermal stress at 42 °C (Fig. S3), it is likely that Ded1 localizes to the SGs under severe ethanol stress.

Hsp104 and Hsp70 associate with heat-induced SGs, and facilitate SG disassembly and translation re-initiation during recovery from severe heat shock (56, 57). It has also been reported that, in mammalian cells, chaperones prevent the formation of aberrant SGs and promote the disassembly of SGs when the stress subsides (58, 59). Therefore, we examined whether Ded1 colocalizes with Hsp104, which functions as a disaggregase in the bi-chaperone system for the dissolution of denatured protein aggregates with Hsp70 and co-chaperone(s) in yeast cells (60, 61, 62). As shown in Figure 4*B*, Ded1-GFP colocalized with Hsp104-mRFP, forming cytoplasmic granules under severe ethanol stress and thermal stress at 42 °C. In the cells pretreated with 6% ethanol, Ded1-GFP and Hsp104-mRFP also initially colocalized, but Ded1-GFP dispersed with time under subsequent 10% ethanol, whereas Hsp104-mRFP continued to form granules for a long time (>180 min). These results imply that Hsp104 may play a role in the disaggregation of Ded1 and translation re-initiation in the pretreated cells under severe ethanol stress.

### *fes1Δhsp104Δ* cells fail to regenerate Ded1 and recover translational activity

**Figure 5:**
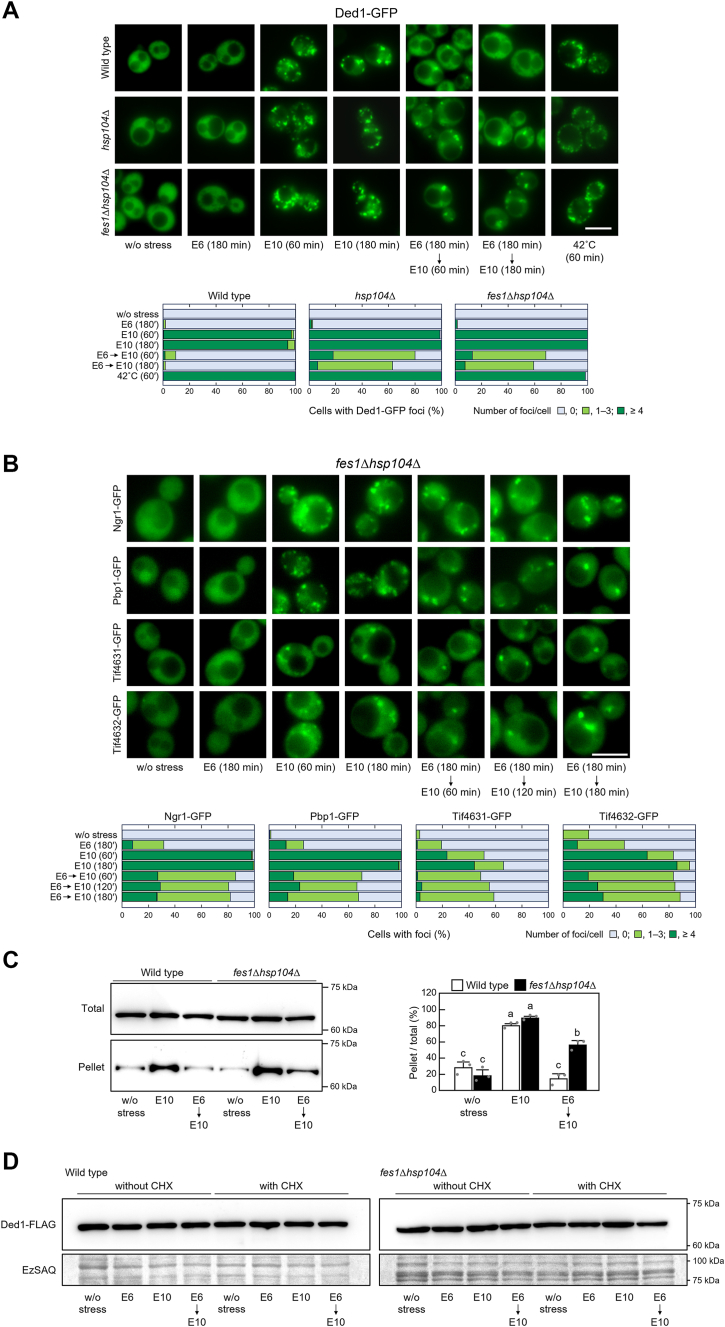
**Bi-chaperone system is involved in the regeneration of Ded1.***A*, intracellular localization of Ded1-GFP was examined in yeast cells under the stress conditions indicated. *B*, formation and disassembly of SGs in *fes1*Δ*hsp104*Δ cells, which are isogenic to wild-type cells (BY4742), were examined using GFP-tagged Ngr1, Pbp1, Tif4631, and Tif4632. Representative images are shown in the *upper panels*, and quantified data are shown in the *lower panels*. Experiments were repeated three times and more than 300 cells in total were examined. Scale bar, 5 μm. *C*, changes in the levels of Ded1 aggregates were examined *via* western blotting. Yeast cells were pre-exposed or not pre-exposed to mild ethanol stress (6% v/v, E6) for 180 min and then exposed to severe ethanol stress (10% v/v, E10) for 180 min. The quantified data are presented in the *right panel* (mean ± S.D., *n* = 3). Different letters indicate statistically significant differences (*p* < 0.01, ANOVA with post-hoc Tukey’s test). *D*, significant degradation of Ded1 was not induced under severe ethanol stress. To monitor the turnover of Ded1-FLAG under severe ethanol stress, yeast cells expressing Ded1-FLAG were subjected to cycloheximide (CHX)-chase assay. Cells were treated with ethanol stress with or without 200 μg/ml CHX.

Since Hsp104 was inferred to be involved in the recovery of translational activity in the pretreated cells, we next investigated the ethanol stress response of *fes1*Δ*hsp104*Δ cells. Fes1 is a nucleotide exchange factor for Hsp70 (Ssa1–4) and its deletion reduces the cellular Hsp70 capacity (63). Thus, *fes1*Δ*hsp104*Δ cells possess a greatly reduced ability to disaggregate and regenerate aggregated proteins through the bi-chaperone system (62). As shown in Figure 5*A*, in *fes1*Δ*hsp104*Δ cells and wild-type cells, the formation of Ded1 aggregates occurred under 10% ethanol but not under non-stressed conditions. Unlike wild-type cells, *fes1*Δ*hsp104*Δ cells pretreated with 6% ethanol retained Ded1-GFP granules after 180 min of exposure to 10% ethanol (Fig. 5*A*). Furthermore, *fes1*Δ*hsp104*Δ cells induced SG formation, albeit at low frequency, during the pretreatment with 6% ethanol and failed to induce SG disassembly under 10% ethanol stress even after the pretreatment (Fig. 5*B*). When the Ded1 status under 10% ethanol stress was examined, aggregated Ded1 appeared to resolubilize in the pretreated wild-type cells, while Ded1 remained aggregated in *fes1*Δ*hsp104*Δ cells (Fig. 5*C*). Because Ded1 levels remained nearly constant under ethanol stress with or without cycloheximide (Fig. 5*D*), Ded1 did not appear to be newly synthesized or degraded in large amounts in pretreated cells. Rather, it is more likely that aggregated Ded1 in pretreated wild-type cells is regenerated through the bi-chaperone system.

**Figure 6:**
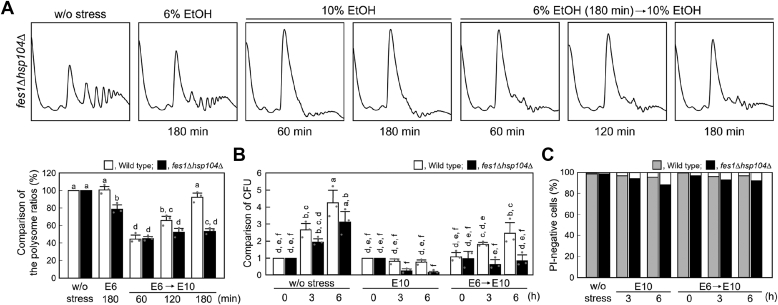
**Insufficient restoration of translational activity in *fes1*Δ*hsp104*Δ cells pre-exposed to mild ethanol stress**. *fes1*Δ*hsp104*Δ cells were pre-exposed or not pre-exposed to mild ethanol stress (6% v/v, E6) for 180 min and then exposed to severe ethanol stress (10% v/v, E10). *A*, the polysome profiles of *fes1*Δ*hsp104*Δ cells under ethanol stress were determined. The polysome ratios under each condition were compared and expressed as a percentage of that of the non-stressed cells (mean ± S.D., *n* = 3). *White bars* indicate the data shown in Figure 1. Different letters indicate statistically significant differences (*p* < 0.05, ANOVA with post-hoc Tukey’s test). *B*, cell proliferation was evaluated using CFU after the stress treatments. The CFU of untreated cells at 0 h was set to a relative value of 1 (mean ± S.D., *n* = 3). White bars are identical to the data in Figure 2*B*. Different letters indicate statistically significant differences (*p* < 0.05, ANOVA with post-hoc Tukey’s test). *C*, propidium iodide (PI) staining was performed to evaluate cell death. One hundred cells under each condition were examined, and experiments were repeated three times independently (300 cells in total were examined) (mean ± S.D.).

Polysome analysis revealed that *fes1*Δ*hsp104*Δ cells maintained almost the same translational activity level as that of wild-type cells under non-stressed conditions (Figs. 1 and 6*A*). However, *fes1*Δ*hsp104*Δ cells showed a significant decrease in polysome ratios after treatment with 6% ethanol (*p* < 0.001 for the significant difference before and after 6% ethanol treatment). Additionally, *fes1*Δ*hsp104*Δ cells pretreated with 6% ethanol failed to fully recover the polysome ratios under subsequent 10% ethanol treatment (Fig. 6*A*). Furthermore, *fes1*Δ*hsp104*Δ cells could not resume proliferation under subsequent 10% ethanol treatment even after the pretreatment with 6% ethanol (Fig. 6*B*). On the other hand, the percentage of propidium iodide (PI)-negative *fes1Δhsp104*Δ cells remained high (>89%) under severe ethanol stress (Fig. 6*C*). These results suggest that the decrease in colony forming units (CFUs) of *fes1Δhsp104*Δ cells under 10% ethanol stress was due to growth arrest rather than cell death. The lower CFU of *fes1*Δ*hsp104*Δ cells under 10% ethanol stress compared to wild-type cells, even after the pretreatment with 6% ethanol, seems also to be due to the lack of translational activity, which prevents recovery of proliferative capacity. Alternatively, *fes1*Δ*hsp104*Δ cells might not have dealt well with the delayed damage caused by ethanol. The slight recovery of CFU in pretreated *fes1*Δ*hsp104*Δ cells was presumably due to factors other than the bi-chaperone system.

**Figure 7:**
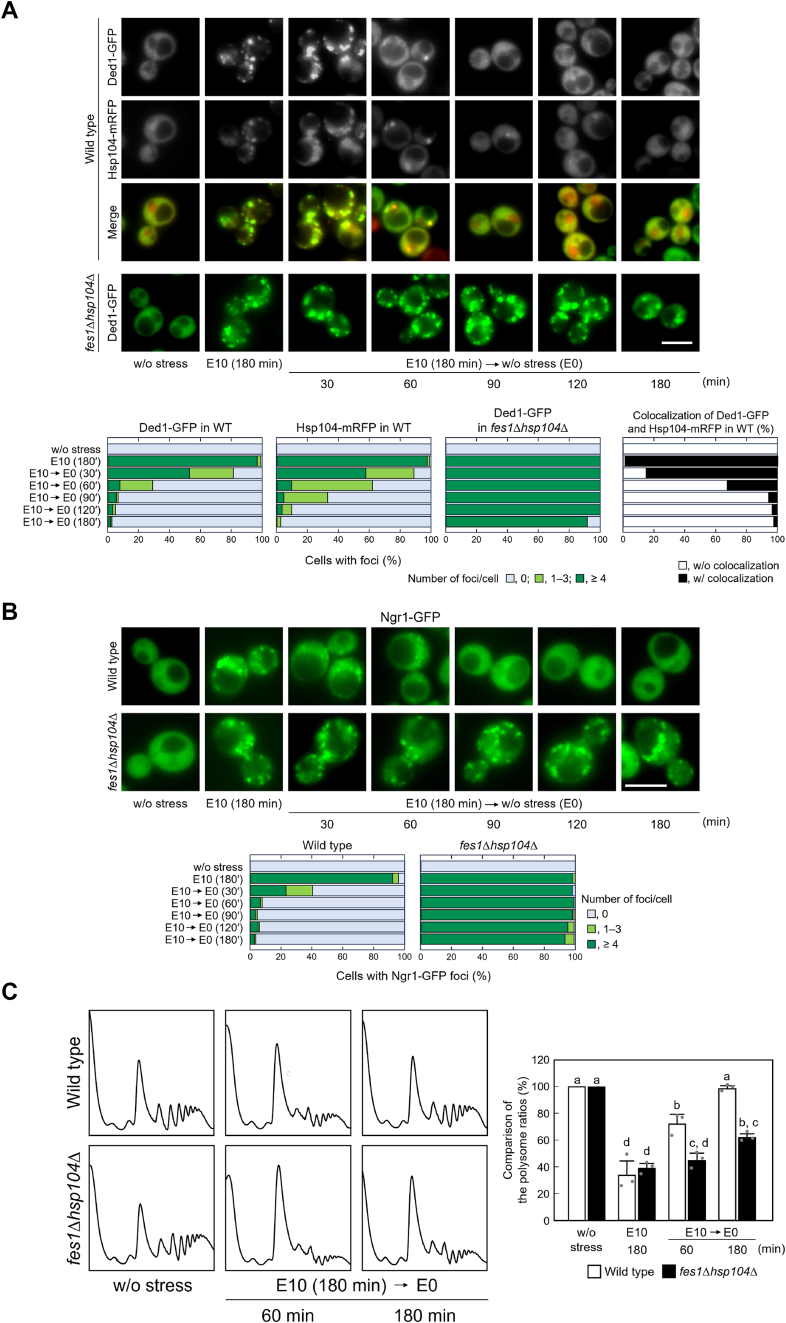
**Insufficient restoration of Ded1 and translational activity in *fes1*Δ*hsp104*Δ cells during the recovery process**. Wild-type cells and *fes1*Δ*hsp104*Δ cells were exposed to severe ethanol stress (10% v/v, E10) for 180 min and then transferred into fresh SD media without ethanol (E0). *A*, the intracellular localization of Ded1-GFP was examined. Colocalization of Ded1-GFP and Hsp104-mRFP in wild-type cells was also examined. Scale bar, 5 μm. *B*, SG formation was examined using GFP-tagged Ngr1. Scale bar, 5 μm. The quantification of cells containing foci is shown in the *lower panel*. The experiments were repeated three times, and more than 300 cells were examined. *C*, the polysome ratios under each condition were compared and expressed as a percentage of that of the non-stressed cells (mean ± S.D., *n* = 3). Different letters indicate statistically significant differences (*p* < 0.01, ANOVA with post-hoc Tukey’s test).

During recovery from severe ethanol stress, *fes1*Δ*hsp104*Δ cells showed a delay in the disaggregation of Ded1 and SGs compared to that shown by wild-type cells (Fig. 7, *A* and *B*). *fes1*Δ*hsp104*Δ cells maintained Ded1 aggregates and SGs even 180 min after the transition from 10% ethanol stress to non-stressed conditions (0% ethanol), whereas wild-type cells lost these aggregates almost completely (Fig. 7, *A* and *B*). In addition, there was a significant difference in the recovery of translational activity between the wild-type and *fes1*Δ*hsp104*Δ cells (Fig. 7*C*). These results suggest that the bi-chaperone system is involved in the rapid reconstruction of translational activity during recovery from severe ethanol stress.

## Discussion

In this study, we demonstrated that Ded1 aggregation, not eIF2α phosphorylation, is involved in the translation repression caused by severe ethanol stress. This finding indicates that translation repression induced by dysfunctional Ded1 is not limited to heat shock and glucose depletion (41, 42). Ded1 has been reported to be a superaggregator that is readily denatured and aggregates following heat shock (64). Similar to heat shock, 10% ethanol damages proteins and causes the accumulation of insoluble proteins in yeast cells (9). Therefore, it seems reasonable to assume that Ded1 is denatured under severe ethanol stress and easily dissociates from mRNA and aggregates. The aggregation-prone characteristic of Ded1 may provide a simple and definitive mechanism for rapid translation repression *via* its dysfunction. Since DDX3, a human homolog of Ded1, also aggregates and is involved in translation repression under stress conditions (65, 66), dysfunction of Ded1/DDX3 may be one of the major causes of translation repression under various stress conditions, and is well-conserved across species.

This study also demonstrated the resilience of yeast translational activity to severe ethanol stress. The translational activity was restored under 10% ethanol stress in yeast cells pretreated with 6% ethanol but remained repressed in yeast cells directly exposed to 10% ethanol. In parallel with the recovery of translational activity, the disappearance of Ded1 aggregates was observed under 10% ethanol stress in the pretreated wild-type cells. For yeast cells to adapt to the subsequent severe ethanol stress and resume growth, maintaining Ded1 activity seems crucial for the expressing housekeeping genes (67). These results provided new insight into the outstanding ability of yeast cells to cope with severe ethanol stress.

Ded1 is degraded *via* an autophagic pathway upon TORC1 inhibition by rapamycin (40). However, it is unlikely that Ded1 degradation is induced under severe ethanol stress since macroautophagy and proteasome activity are suppressed by severe ethanol stress (9, 68). Indeed, a cycloheximide-chase assay (69) confirmed that no significant degradation of Ded1 occurred under ethanol stress conditions (Fig. 5*D*). Aggregated Ded1 is likely regenerated by the bi-chaperone system, rather than being degraded and eliminated.

The bi-chaperone system consisting of Hsp104 and Hsp70 plays a key role in the yeast protein quality control (PQC) system for the regeneration of diverse aggregated proteins and disassembly of SGs (57, 62, 70, 71). *fes1*Δ*hsp104*Δ cells could not regenerate Ded1 and restore translational activity under severe ethanol stress, even after pretreatment with 6% ethanol. These findings indicated that the bi-chaperone system is also involved in regenerating Ded1 and preventing the accumulation of Ded1 aggregates. We previously confirmed that pretreatment with 6% ethanol enhances the protein levels of various PQC-related factors including Hsp104, Hsp70 (Ssa3), and Fes1 (10). These results indicate that the bi-chaperone system is upregulated in the pretreated cells, and that the enhanced bi-chaperone system is likely essential for Ded1 regeneration in the presence of 10% ethanol. The basal level activity of the bi-chaperone system was unable to regenerate Ded1 aggregates upon direct exposure to 10% ethanol; thus, upregulation of the bi-chaperone system seems to be necessary to regenerate Ded1 and restore translational activity under severe ethanol stress. Since aggregated proteins are not as abundant under laboratory conditions without stress (9, 10, 64), the activity of the bi-chaperone system might be maintained at a low level in cells under non-stressed conditions. Thus, its basal activity may not have been sufficient to regenerate the aggregated Ded1 upon acute 10% ethanol stress. Activation of the bi-chaperone system may be crucial in providing resilience not only to Ded1 but also to the entire translation machinery under severe ethanol stress. It is speculated that degenerated translation-related factors other than Ded1 must also be disaggregated to get recovery under severe ethanol stress. Various translation-related factors including Tif5 and Tif34 have been reported to be prone to aggregation by heat shock (64), and these factors may also be degenerated by severe ethanol stress and regenerated through the bi-chaperone system. This possibility needs to be investigated further in the future. Since mild thermal stress at 37 °C also enhances protein levels of Hsp104, Hsp70 (Ssa3), and Fes1 (10), cells pretreated at 37 °C are expected to restore translational activity with gradual disaggregation of Ded1 under subsequent severe ethanol stress, although confirmation is needed.

The RNA helicase activity of Ded1 is critical for the efficient translation initiation of mRNAs with long and highly structured 5′-untranslated regions (UTRs), and housekeeping transcripts are generally known to have long, highly structured 5′-UTRs (39, 42, 43, 72, 73). However, most stress-response transcripts, such as *HSP* mRNAs have short and unstructured 5′-UTRs and can evade translation repression by Ded1 inactivation (42). Heat shock and glucose depletion are well known to cause the synthesis of various HSPs (30, 74), and severe ethanol stress is also known to induce preferential translation of *HSP42*, *HSP78*, *HSP104*, and *BTN2* (10, 11, 19). This and previous studies demonstrated that Ded1 forms aggregates under these three conditions (42, 44) (Fig. 4*A*). Synthesis of these stress-response proteins is likely to occur in a Ded1-independent manner.

The preferential translation of stress-response transcripts may be facilitated by inhibiting the translation of housekeeping transcripts *via* Ded1 aggregation. To prioritize the translation of stress-response transcripts under stress conditions, it might be more effective to suppress the translation of housekeeping transcripts *via* Ded1 inactivation rather than to repress the global Cap-dependent translation by reducing the supply of the ternary complex *via* eIF2α phosphorylation. Alternatively, to respond appropriately to various stress conditions, using different methods of translation inhibition may be important. Indeed, similar to glucose depletion (16, 54, 75), severe ethanol stress only slightly induced phosphorylation of eIF2α within 180 min (Fig. 3). Severe heat shock also inhibits translational activity in a Gcn2-independent manner (23, 54, 76). In contrast, no Ded1-aggregation or Ded1-dissociation from the ribosomes was observed during translation repression by nitrogen starvation, which induces phosphorylation of eIF2α (52, 77) (Figs. 3 and 4).

Severe ethanol stress caused aggregation of Tif3 (eIF4B) in addition to aggregation of Ded1. Tif3 is also prone to aggregation under heat shock (64) and cooperates with Ded1 to promote the translation of mRNAs containing long, structured 5′-UTRs (72). The rapid aggregation of both Ded1 and Tif3 would ensure the repression of the translation of housekeeping transcripts. In contrast, severe ethanol stress did not induce eIF4A (Tif1 and Tif2) aggregation. However, the possibility that eIF4A dissociates from mRNA and loses its function in the translation initiation stage under severe ethanol stress cannot be ruled out at this point, because eIF4A aggregates were not observed even under heat shock conditions at 42 °C, which has been reported to dissociate eIF4A from mRNA (41).

Recent studies have suggested that Ded1 functions as a stress sensor that responds directly to sudden environmental changes and exerts multiple effects during the cellular stress response (42, 67), and our results are consistent with this idea. Additionally, the bi-chaperone system, which is required for Ded1 regeneration, may also be crucial for the resilience of yeast to various stresses. Since DDX3/Ded1 dysfunction is implicated in medulloblastoma (65, 78), the protection and recovery of DDX3/Ded1 function by the bi-chaperone system would be an interesting research subject from the perspectives of pathology and cancer therapy.

## Experimental procedures

### Yeast strains, media, and stress treatment

The parental wild-type strain BY4742 (*MAT*α *his3*Δ*1 ura3*Δ*0 leu2*Δ*0 lys2*Δ*0*) and its isogenic knockout mutants (*gcn2*Δ and *hsp104*Δ) were purchased from Open Biosystems. The double knockout mutant *fes1*Δ*hsp104*Δ, which is also isogenic to BY4742, was established as previously described (10). Yeast cells were cultured in synthetic defined (SD) medium (2% glucose, 0.67% yeast nitrogen base w/o amino acids, 20 mg/L uracil, 30 mg/L L-lysine HCl, 100 mg/L L-leucine, and 20 mg/L L-histidine HCl) with reciprocal shaking (120 rpm) at 28 °C, and exponentially growing cells were harvested at OD_600_ of 0.5 to 0.6. Stress treatment procedures have been previously described (10). To determine cell proliferation activity, cells subjected to severe ethanol stress in SD medium were diluted, plated on YPD plates (2% glucose, 1% yeast extract, 2% peptone, and 2% agar), and incubated at 28 °C for 48 h. The number of colonies formed on YPD plates was counted to calculate CFU. Propidium iodide (PI) staining was performed using the method described by Davey and Hexley (79).

### Plasmids

All integrative plasmids for expressing GFP-tagged proteins (YIp-*GFP* series) were constructed by cloning a part of the open reading frame (ORF) of each gene, which was amplified *via* polymerase chain reaction (PCR) using the genomic DNA of BY4742 as the template and the primer sets of F1/R1 (Table 1), into pJK67 (80). YIp-*SUI2-FLAG* and YIp-*DED1-FLAG* were constructed by replacing the *GFP* region of YIp-*SUI2-GFP* and YIp-*DED1-GFP* with a DNA fragment encoding the FLAG tag sequence, stop codon, and the 3′-flanking region of *SUI2* or *DED1*, which was amplified with the primers *SUI2*-F2/R2 and *DED1-*F2/R2, respectively. YIp-*HSP104-mRFP* and YIp-*DED1-mRFP* were constructed by cloning a part of ORF of *HSP104* or *DED1*, which were amplified *via* PCR with the primers *HSP104*-F3/R3 and *DED1-*F3/R3, respectively, into YIp-*LSG1-mRFP* (10). YIp-*GCN4*-*FLAG* was constructed by cloning a part of the ORF with the FLAG tag sequence, stop codon, and the 3′-flanking region of *GCN4*, which was amplified by overlap extension PCR with the primers *GCN4*-F1/R1/F2/R2 (81). YIp-*NGR1-GFP*, YIp-*PBP1-GFP*, and YIp-*TIF4632-GFP* were constructed as described previously (29, 49). The plasmids with the *HIS3* marker were constructed by cloning the regions encoding the GFP- or FLAG-tagged genes to be analyzed into pRS303 (82). Each gene was introduced at its chromosomal locus, and transcription of each gene was controlled by its native promoter.

**Table 1:** List of primers used in plasmid construction

Name	Sequence
*TIF1*-F1	5′-ACCGCGGTGGCGGCCGCTCTAGACGTAGATTCAGAACTG ACAAGATCAAGATG-3′
*TIF1*-R1	5′-CTCCTTTGCTAGCCATAGCTCGAGAGTTCAACAAAGTAG CGATGTCGGATGGCA-3′
*TIF2*-F1	5′-CGCGGTGGCGGCCGCTCTAGATGAAGCTGATGAAATGT TGTCTTC-3′
*TIF2*-R1	5′-CTTTGCTAGCCATAGCTCGAGAGTTCAACAAGGTAGCA ATGTCGG-3′
*TIF3*-F1	5′-ACCGCGGTGGCGGCCGCTCTAGATTAGACCCTGCTTTGG GGGGCGGTTCTTCC-3′
*TIF3*-R1	5′-CTCCTTTGCTAGCCATAGCTCGAGATTTCTTACCAACAA CTTCCCAATTGTCACC-3′
*TIF4631-*F1	5′-CTTGTTCCAAGAGCTCATAGGTGGGTGCCA-3′
*TIF4631-*R1	5′-ATGATCTATTCTCGAGACTCTTCGTCATCA-3′
*DED1*-F1	5′- ACCGCGGTGGCGGCCGCTCTAGATGAAGCTGATAGAAT GTTGGATATGGGTTT-3′
*DED1*-R1	5′-CTCCTTTGCTAGCCATAGCTCGAGACCACCAAGAAGAG TTGTTTGAACCACCGCT -3′
*DED1-*F2	5′-ACTCTTCTTGGTGGTCTCGAGCTGACTACAAGGATGACG ATGACAAGTGATTTCAGACAAACTAGGGTGAGGATTC-3′
*DED1-*R2	5′-AGGGAACAAAAGCTGGGTACCGCAAAGGCATTTTATAG TACAATTG-3′
*DED1-*F3	5′-CGCGGTGGCGGCCGCTCTAGCTGATAGAATGTTGGATAT GGGTTTCGAAC-3′
*DED1-*R3	5′-CGTCCTCGGAGGAGGCTCGAGACCACCAAGAAGAGTTG TTTGAACCACCG-3′
*SUI2*-F1	5′-CCGCGGTGGCGGCCGCTCTAGATCCAGCAGATTGCTGAA ATGGGTGC-3′
*SUI2*-R1	5′-CTTCTCCTTTGCTAGCCATAGCTCGAGACTCGTCGTCTG ACTCATCCTCATCGTCTTC-3′
*SUI2-*F2	5′-GAGTCAGACGACGAGTCTCGAGCTGACTACAAGGATGA CGATGACAAGTAATCATTGCCGCGCCTAATTTTTCTAGGT-3′
*SUI2-*R2	5′-AGGGAACAAAAGCTGGGTACCGAGCCTGCATGTGTTGC TGGACGTATTGAC-3′
*HSP104-*F3	5′-CTATAGGGCGAATTGGAGCTCCGGTTCCGGTAAAACTG AATTGG-3′
*HSP104-*R3	5′-CGTCCTCGGAGGAGGCTCGAGAATCTAGGTCATCATCAA TTTCCA-3′
*GCN4*-F1	5′-CTATAGGGCGAATTGGAGCTCTTCCACAAACGGCAACTG CACCTGA-3′
*GCN4*-R1	5′-CTTGTCATCGTCATCCTTGTAGTCGCGTTCGCCAACTAAT TTCTTTAA-3′
*GCN4*-F2	5′-GACTACAAGGATGACGATGACAAGTGATTTCATTTACCT TTTATTTTAT-3′
*GCN4*-R2	5′-GGTACCGGGCCCCCCCTCGAGGATTCCATTTTGAGGATTC CTATATC-3′

### Polysome analysis

Polysome analysis was performed using the method reported by Inada and Aiba (83) using a gradient master and fractionator (107–201 M and 152–002; BioComp Instruments). The polysome ratio was calculated as the percentage of the area under polysomal ribosome peaks relative to that under total ribosome peaks (84).

### Western blot analysis

To monitor levels of phosphorylated eIF2α (Sui2), anti-phospho-EIF2S1 (Ser52) polyclonal antibody (#44–728G; Thermo Fisher Scientific) and horse radish peroxidase-linked anti-rabbit IgG antibody (406401; BioLegend) were used. The levels of FLAG-tagged Ded1, Sui2, and Gcn4 were analyzed *via* western blotting using an anti-FLAG M2 antibody (F1804; Sigma-Aldrich) and horse radish peroxidase-linked anti-mouse IgG antibody (7076S; Cell Signaling Technology). Ded1 turnover was examined using a cycloheximide-chase assay (76). The aggregate sedimentation assay was performed to monitor the levels of aggregated Ded1. Collected cells were disrupted with glass beads in lysis buffer (50 mM potassium phosphate buffer [pH 6.8], 1 mM ethylenediaminetetraacetic acid, 5% glycerol, and 1× Protease inhibitor [Roth]) to prepare the cell-free extract. Aggregated Ded1 protein was collected *via* centrifugation of the cell-free extract at 16,000*g* for 20 min at 4 °C and then resuspended in urea buffer (50 mM Tris-HCl [pH 7.5], 6.0 M urea, and 5% sodium dodecyl sulphate). The bands on the western blots were quantified using the ImageJ software (http://imagej.nih.gov/ij/) and normalized to total protein levels or EzStainAQua MEM (EzSAQ) staining (WSE-7160; ATTO Corporation).

### Fluorescent microscopic analysis

An IX83 microscope system (Olympus) was used for fluorescence microscopy analysis. The cells were observed immediately after stress treatment without fixation.

### Statistical analysis

Statistical significance was evaluated using one-way ANOVA with Dunnett’s or Tukey's post-hoc test. Data are expressed as the mean ± standard deviation (S.D.) (*n* = 3).

## Data availability

All relevant data can be found within the article and its supporting information. Images, yeast strains, and plasmids are available upon request.

## Supporting information

This article contains supporting information (Figs. S1–S3).

## Conflict of interest

The authors declare that they have no conflicts of interest with the contents of this article.
