# Supplementary material for: Contribution of the yeast bi-chaperone system in the restoration of the RNA helicase Ded1 and translational activity under severe ethanol stress
Source: J Biol Chem. 2023 Nov 17;299(12):105472. doi: 10.1016/j.jbc.2023.105472 (PMC10746526; doi:10.1016/j.jbc.2023.105472)
Supplement: Supporting information [file mmc1.pdf]

Supporting Information

Contribution of the yeast bi-chaperone system in the restoration of the RNA helicase Ded1 and translational activity under severe ethanol stress

Ryoko Ando[1], Yu Ishikawa[1], Yoshiaki Kamada[2], Shingo Izawa[1,#]

[1]*Graduate School of Science and Technology, Kyoto Institute of Technology, Matsugasaki, Sakyo-ku, Kyoto 606-8585, Kyoto, Japan*

[2]*National Institute for Basic Biology, Okazaki, 444-8585, Aichi, Japan*

[#]**Correspondence**: Shingo Izawa, E-mail: thioredoxin@kit.ac.jp

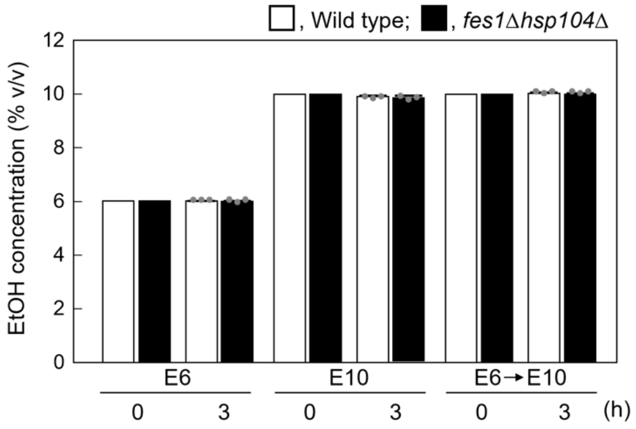

Figure S1. Ethanol concentrations in the cultures.

Cells were pre-exposed or not pre-exposed to mild ethanol stress (6% v/v, E6) for 3 h and then exposed to severe ethanol stress (10% v/v, E10). The ethanol concentrations in the cultures after stress treatment were measured using gas chromatography (AL-2; Riken Keiki Co., Tokyo, Japan). No significant decrease was detected after 3 h of stress treatment (mean ± S.D., *n* = 3).

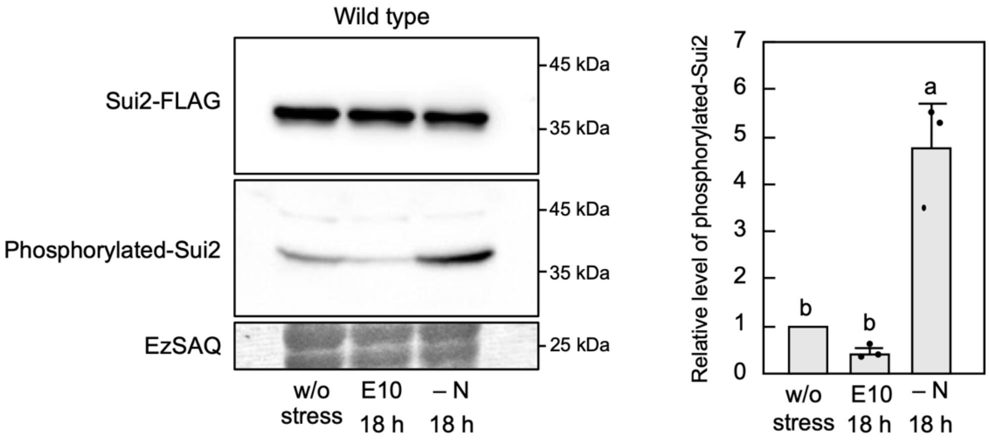

Figure S2. Phosphorylation of eIF2α (Sui2) is not induced even under long-term severe ethanol stress.

Yeast cells expressing Sui2-FLAG were subjected to severe ethanol stress (10% v/v, E10) or nitrogen starvation stress (–N) for 18 h. The levels of FLAG-tagged Sui2 and phosphorylated Sui2 were assayed by western blotting. EzSAQ staining was performed to confirm equal loading and the transfer of all proteins. Phosphorylated Sui2 levels were quantified using the ImageJ software. The phosphorylated Sui2 level of cells w/o stress treatment was set to a relative value of 1 (mean ± S.D., n =3). Different letters indicate statistically significant differences ($p < 0.001$, ANOVA with post hoc Tukey's test).

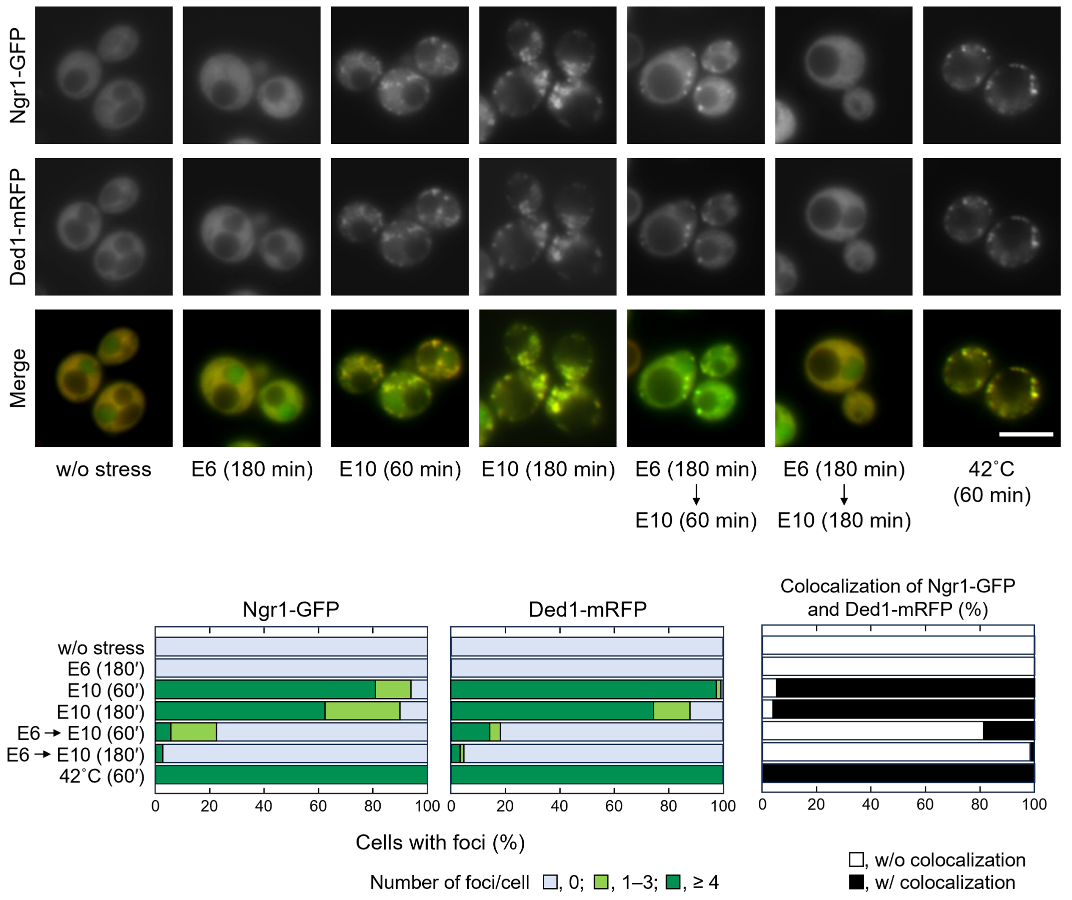

Figure S3. Ded1 is a component of stress granules formed under severe ethanol stress and heat shock at 42°C.

Colocalization of Ded1 and Ngr1 was monitored in wild-type cells expressing Ded1-mRFP and Ngr1-GFP under the stress conditions indicated. Representative images are shown in the upper panels, and quantification of cells containing foci is shown in the lower panels. The experiments were repeated three times, and more than 300 cells in total were examined. E6 and E10 represent 6% and 10% (v/v) ethanol stress, respectively. Scale bar, 5 μm.
